# A Review of the Environmental Trigger and Transmission Components for Prediction of Cholera

**DOI:** 10.3390/tropicalmed6030147

**Published:** 2021-08-05

**Authors:** Moiz Usmani, Kyle D. Brumfield, Yusuf Jamal, Anwar Huq, Rita R. Colwell, Antarpreet Jutla

**Affiliations:** 1 Geohealth and Hydrology Laboratory, Department of Environmental Engineering Sciences, University of Florida, Gainesville, FL 32603, USA; moiz.usmani@ufl.edu (M.U.); yjamal@ufl.edu (Y.J.); antar.jutla@essie.ufl.edu (A.J.); 2 Maryland Pathogen Research Institute, University of Maryland, College Park, MD 20742, USA; kbrum@umd.edu (K.D.B.); huq@umd.edu (A.H.); 3 University of Maryland Institute for Advanced Computer Studies, University of Maryland, College Park, MD 20742, USA

**Keywords:** environmental parameters, cholera, *Vibrio cholerae*, trigger, transmission, prediction

## Abstract

Climate variables influence the occurrence, growth, and distribution of *Vibrio cholerae* in the aquatic environment. Together with socio-economic factors, these variables affect the incidence and intensity of cholera outbreaks. The current pandemic of cholera began in the 1960s, and millions of cholera cases are reported each year globally. Hence, cholera remains a significant health challenge, notably where human vulnerability intersects with changes in hydrological and environmental processes. Cholera outbreaks may be epidemic or endemic, the mode of which is governed by trigger and transmission components that control the outbreak and spread of the disease, respectively. Traditional cholera risk assessment models, namely compartmental susceptible-exposed-infected-recovered (SEIR) type models, have been used to determine the predictive spread of cholera through the fecal–oral route in human populations. However, these models often fail to capture modes of infection via indirect routes, such as pathogen movement in the environment and heterogeneities relevant to disease transmission. Conversely, other models that rely solely on variability of selected environmental factors (i.e., examine only triggers) have accomplished real-time outbreak prediction but fail to capture the transmission of cholera within impacted populations. Since the mode of cholera outbreaks can transition from epidemic to endemic, a comprehensive transmission model is needed to achieve timely and reliable prediction with respect to quantitative environmental risk. Here, we discuss progression of the trigger module associated with both epidemic and endemic cholera, in the context of the autochthonous aquatic nature of the causative agent of cholera, *V. cholerae*, as well as disease prediction.

## 1. Introduction

Cholera is transmitted primarily by ingestion of contaminated water containing the bacterium *Vibrio cholerae* and has plagued the world for centuries. The ongoing cholera pandemic, the seventh, which started in the 1960s, continues to claim millions of victims every year and is considered the world’s longest-running pandemic [1,2,3].This acute diarrheal disease remains one of the most significant public health burdens in many regions globally, notably in Latin America, sub-Saharan Africa, and Southern Asia [4,5], where an estimated one million cases are reported every year [6]. The World Health Organization estimates that up to four million reported cholera cases occur across the world annually [4]. However, the actual number of cholera cases is likely much higher as many cases go unreported, especially in developing countries.

In recent years, cholera outbreaks have originated primarily in coastal areas [7,8]. The disease is prevalent in parts of the world where human vulnerability (i.e., lack of access to clean water and appropriate sanitation) intersects with changes in hydrological and environmental processes, which provide conditions favorable for the occurrence and growth of *V. cholerae* in the aquatic environment. Furthermore, massive cholera outbreaks are often associated with natural and anthropogenic disasters. A recent example is one of the largest cholera outbreaks in 2016 during the months following Hurricane Matthew [9], which lashed rains over the southwestern coast of Haiti. Damage to water, sanitation, and hygiene (WASH) infrastructure coupled with elevated air temperatures and above-average rainfall promoted exposure of the population to contaminated water. An outbreak of cholera was reported subsequently.

Throughout history, during periods of active conflict and raging wars, infectious diseases have claimed more lives than actual war-induced injuries [10]. Since March 2015, Yemen, a coastal Middle Eastern country, has experienced surges of violent civil unrest. In October 2016, Yemen reported the first of a series of sporadic cholera outbreaks. After the initial reports, the number of cases declined briefly for a few months until the WASH infrastructure failed, resulting in a severe spike in the number of reported cholera cases. The resurgence of the disease and continued environmental exposure of the population proved disastrous to public health. By the end of 2017, Yemen was experiencing the largest cholera outbreak in recorded history [11], which ultimately accounted for an estimated 80% of the globally reported cholera cases that had been recorded since 2015 [12]. While natural disasters can be catalytic for cholera, the Yemen cholera outbreak demonstrates the enormous potential for an anthropogenic catastrophe to affect public health similarly and perhaps even more devastatingly.

Cholera occurs predominantly in two forms: epidemic, characterized either by the sporadic or rampant occurrence of cases in an outbreak; and endemic, defined as cases occurring annually at a continuous level, often with distinct seasonal peaks in the number of cases. Data from epidemiological surveillance suggest that the Yemen cholera outbreak began in the epidemic mode [13]. The dominant hypothesis for epidemic cholera is related to conditions when the air temperature is suddenly anomalously high and excess rainfall occurs with insufficient and/or damaged WASH infrastructure in the region. Human populations will then be at higher risk of exposure to cholera bacteria, hence the disease [14,15]. Per contra, endemic cholera has been shown to occur in a region where *V. cholerae* is constant, even at low abundance, and circulating in the aquatic environment (e.g., rivers, estuaries, and coastal aquatic ecosystems providing conditions favorable for the bacterium). Often, environmental factors influencing endemic cholera will result in cyclical or seasonal recurrence of the disease [16,17]. A sustained epidemic mode of cholera can evolve to become endemic in regions, with the potential for enhanced and continued exposure to, and transmission of, *V. cholerae* [18]. From our previous research [2,19,20,21,22,23], it is understood that *V. cholerae* ecology must be viewed in the context of its natural aquatic environment and a changing climate driving cholera as a potential re-emerging infectious disease.

The dominant forms of cholera (epidemic and endemic) are guided by two components that are key to a disease outbreak, namely trigger and transmission. The trigger module (TM) comprises those mechanisms that support the growth, multiplication, persistence, and distribution of *V. cholerae* in the environment. That is, when TM indicates conditions are favorable for the high abundance of the bacterium and is coincident with insufficient WASH infrastructure, there will be increased interaction between *V. cholerae* and the human population. Following a prevailing TM, the transmission component (TrM) comprises pathways by which an outbreak of cholera will occur and engages complex interactions between humans and contaminated water. The foundational theory of TrM is that humans can accelerate the spread of cholera via intestinal colonization and shedding of cholera bacteria into the environment, thereby contaminating drinking water systems [18]. Given favorable environmental conditions, the bacterium multiplies and can infect a population through the fecal–environmental–oral transmission route. Here, we discuss progression of the TM underlying epidemic cholera, and the TrM associated with both epidemic and endemic cholera, in the context of *V. cholerae* as a bacterium autochthonous to the aquatic habitat and prediction of cholera in the human population.

## 2. *Vibrio cholerae* and Its Natural Habitat

*Vibrio cholerae*, the causative agent of the acute diarrheal disease cholera, is a Gram-negative bacterium native to the aquatic environment. Historically, detection of *V. cholerae* was achieved by determining its presence clinically during cholera outbreaks [24]. However, before the advent of epifluorescent microscopy [25,26] and molecular markers [27,28,29,30,31], detection of the presence of *V. cholerae* in the environment was accomplished by employing culture-based techniques [32]. Such investigations significantly underestimated *V. cholerae* populations in the environment, namely because the bacterium can enter a viable but non-culturable (VBNC) state [33]. In the environment and between outbreaks, when environmental conditions are unfavorable for growth and reproduction, the VBNC state allows the bacterium to become metabolically dormant [34,35]. When environmental conditions again become favorable, VBNC cells regain cultivability, having retained virulence potential [36,37]. Furthermore, *V. cholerae* attaches to zooplankton by switching from motile to biofilm lifestyles, which enhances long-term survivability of the bacterium in the environment [38]. Zooplankton, namely copepods, feed on components of the phytoplankton population. Hence, an association between the occurrence of copepods and phytoplankton blooms has been observed [19]. In nutrient-rich water, the increase in the phytoplankton population followed by a zooplankton bloom results in an abundance of *V. cholerae* in coastal waters [16,22]. Because a single copepod can carry up to 10^4^ *V. cholerae* cells [19,39], ingestion of untreated drinking water containing a small number of copepods can increase the risk of infection significantly [40,41,42]. Thus, copepods are a major host and vector of disease. *V. cholerae* has also been observed at high densities attached to abiotic substrates, such as sediment, and associated with various aquatic organisms (e.g., crustaceans, arthropods, fishes, waterfowl, and aquatic plants) [20]. Conversely, in the environment, *V. cholerae,* in association with phages and protozoa, can form antagonistic relationships that reduce microbial populations and shape evolution [20,43].

*V. cholerae* shares many genotypic and phenotypic characteristics with other bacterial taxa, namely Enterobacteriaceae, and toxigenic strains of *V. cholerae* have acquired the ability to produce cholera toxin, a primary virulence factor, via horizontal gene transfer mediated by a lysogenic bacteriophage [44]. The presence and broad distribution of its virulence genes in the environment have been well documented, and such genes that play a role in the pathogenicity of *V. cholerae* for humans may, at the same time, have environmental relevance (e.g., allowing for metabolic processes, establishing symbiosis, and/or modulating predator/prey relationships in the natural aquatic environment) [45,46,47]. In the environment, novel phylogenetic lineages of *V. cholerae* have emerged, carrying mutations potentially involved in adapting to aquatic ecosystems [48,49,50]. Environmental factors, such as the presence of chitin and/or nutrient limitation, can influence horizontal gene transfer [51]. Because many environmental *V. cholerae* isolates have been shown to encode various virulence factors [52] and genetic mutations, some of which have the potential to alter virulence factor production [53], horizontally acquire additional pathogenicity genes [54,55], and even undergo serogroup conversion [55], it is important to determine the total number of *V. cholerae* present in given samples.

Changes in the aquatic environment can have an impact on the intensity of a cholera outbreak [2,23,56,57,58], and seasonal outbreaks occur annually in regions where the disease is endemic [16,17,59,60,61]. During outbreaks, the reported number of cholera cases generally peaks during warmer months of the year, notably in Latin America and Africa, but bi-modal peaks are typical in the Bengal Delta region, related to the hydroclimatic influence on the environment in which the bacterium occurs [62]. In Northern Europe and the Atlantic coast of the United States, heatwaves and warming sea temperatures (up to ~1.5 °C over the past half-century) have been associated with long-term increases in abundance of certain pathogenic *Vibrio* spp., namely *V. cholerae*, *V. parahaemolyticus*, and *V. vulnificus* [21]. While it is worth noting that other *Vibrio* spp., such as *Vibrio splendidus*, express virulence factors at low temperatures [63], observed increases in *Vibrio* spp. abundance in Northern Europe and the US were associated with an unprecedented occurrence of environmentally acquired *Vibrio* infections in the human population [21]. Moreover, a changing climate, namely increased sea temperature, could lead to prolonged seasonal abundance of *V. cholerae,* with profound public health implications [64].

Since *V. cholerae* is autochthonous to the aquatic environment, playing an essential role in nutrient cycling [65,66], cholera cannot be eradicated. Therefore, the ecology of *V. cholerae* must be understood in terms of those environmental parameters that drive cholera, especially as a re-emerging infectious disease and in constructing risk prediction models. Furthermore, early warning systems will be needed to safeguard public health in geographical regions vulnerable to natural disasters such as hurricanes and earthquakes or active conflict, namely social strife or civil war, with resultant damage to safe water and sanitation infrastructure.

## 3. Trigger and Transmission Components for Prediction of Cholera

Traditionally, the spread of cholera has been associated with human activity, notably travel [24] and not hydroclimatic processes. Hydroclimatic processes control the distribution, growth, and incidence of *V. cholerae* in aquatic ecosystems [2] and contribute to genetic diversity and epidemic potential [48]. Therefore, the spread of cholera is a complex function of global travel coupled with climatic processes and the subsequent potential exposure of populations to new spatial and temporal disease outbreaks. Thus, epidemiological research can improve public health interventions aimed at controlling cholera by employing environmental predictive modeling. In 1996, Colwell [2] reported that environmental variables were linked to cholera epidemics and could be evaluated using remote sensing and utilized to develop predictive cholera models. Subsequently, several investigators have confirmed the association of *V. cholerae* with environmental parameters, including sea surface temperature [67,68,69], sea surface height [67,68,69], chlorophyll [23,68,70], precipitation [14,71,72], water storage [73], and salinity [74,75], and suggested their use in cholera risk prediction. Accordingly, a mechanistic understanding of environmental factors in the trigger and transmission of cholera has been developed [9,15,76]. While both TM and TrM are important in understanding the global persistence of cholera, high mortality rates observed in epidemic regions (>3%) compared to the endemic areas (<1%) [15] have caused intervention efforts to focus essentially on the TM of predictive cholera modeling systems.

**Figure 1:**
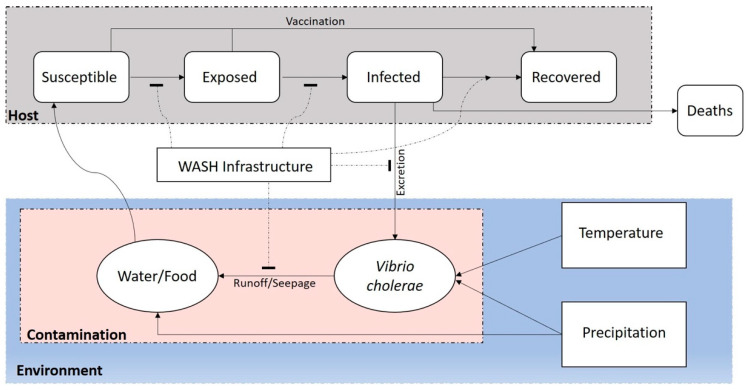
Fundamental cholera susceptible-exposed-infected-recovered (SEIR) transmission model. Susceptible (S) individuals of a population who have been exposed to *Vibrio cholerae* have the potential to acquire the disease from infected (I) individuals. At that point, they also have the potential to become infected (i.e., enter the infectious state), until eventual recovery from the disease (R) or death. Grey shading depicts the SEIR model, which also highlights the potential use of vaccines in curtailing the disease. Water, sanitation, and hygiene (WASH) infrastructure is a critical factor influencing cholera transmission at every stage of the model, from infected to susceptible individuals. Blue represents environmental factors, namely temperature and precipitation, that promote the growth and distribution of *Vibrio cholerae*, the causative agent of cholera, in aquatic reservoirs. Pink shows the potential transmission route from the environment to humans via contaminated food or water containing the *V. cholerae*. Arrow: positive effect; block: negative effect.

In 2013, Jutla et al. [15] proposed the hypothesis for an epidemic cholera trigger risk prediction system whereby anomalously high (defined as more than one standard positive deviation above the long-term average (>30 years)) temperatures followed by anomalously high precipitation, over a period of four weeks, in a region of damaged or compromised WASH infrastructure, facilitated interaction between contaminated water and the human population and comprised an environment favorable for triggering an epidemic cholera outbreak. With this hypothesis, if one or more of the respective conditions are not satisfied, the region has a lower risk of experiencing an outbreak. Initial support of this hypothesis was obtained from analysis of an earthquake that struck Nepal in 2015 [76], and the hypothesis was validated spatially and temporally for several geographic regions, including South Sudan, Cameroon, Zimbabwe, Haiti, Mozambique, Rwanda, Central African Republic, Nepal, and Bangladesh [9,14,15,70,76] Subsequently, the hypothesis was extended to predict the impact of a disaster (natural or anthropogenic) in triggering a cholera outbreak [9,76]. Results showed that natural and anthropogenic disasters that damaged WASH facilities in a region were generally accompanied by high precipitation, collectively making the environment strongly favorable for the growth of *V. cholerae* and increasing human interaction with contaminated water sources. Thus, policy makers and health professionals are now able to use predictive environmental TMs as a tool to prevent, control, and eliminate cholera. It is worth noting, however, that once an outbreak occurs, the TM should be employed in conjunction with TrM to fully capture the progression of cholera in a given region. The transmission component is more broadly useful compared to the TM since TrM largely relates to the mechanism governing the disease dynamics in a human population and is often employed for forecasting the spread of cholera and public health decision-making [77,78]. Many modeling efforts have been made to reduce the disparity between the actual number of cases in a region and the number predicted by the model (i.e., the forecasted number of cases) [72,79,80]. The compartmental model is the most common type of TrM, mainly because it is simple and easy to use [81]. Compartmental models generally divide a given population into three compartments: Susceptible (S), Exposed (E)/Infected (I), and Recovered (R). The four compartments collectively comprise the basic SEIR transmission model [18], a frequently used approach in the epidemiological research domain. Typically, disease dynamics are captured by the rates at which individuals of a population transition between each state (i.e., S, E, I, and R). In Figure 1, we extend the presentation of a basic SIR model to S-E-I-R, accounting for the pathway between the susceptible and exposed populations that have the potential to become infected.

The fundamental theory of the SEIR model is presented as the simultaneous presence of four entities (i.e., S, E, I, and R) required for a cholera outbreak. That is, there must be a sufficient quantity of *V. cholerae* circulating within a population, including a large enough number of susceptible individuals. If any of the four entities is missing, the number of cases in an outbreak is reduced, thereby preventing a sporadic outbreak from becoming epidemic in scale. With respect to the dynamics of cholera, there are cyclic interactions between the human population and the pathogen. The robustness of an SEIR-based TrM relies heavily on the extent to which the module is capable of capturing the interactions. Accordingly, a number of studies have employed various mathematical and biological concepts to modify the basic SEIR model in order to incorporate complex interactions associated with modeling cholera outbreaks [82,83,84,85]. Table 1 summarizes a few key studies utilizing the SEIR model in cholera outbreaks.

More sophisticated SEIR modeling concepts have been proposed with varying constraints of population structure [86], socio-economic factors [86,87], and other critical factors relevant to the transmission dynamics of cholera. Primarily, mathematical sophistication introduced into a SEIR model aims to address the environmental, biological, and behavioral stochasticity inherent in the mechanism of cholera transmission [56]

However, in some cases, rigorous mathematical complexity may impart problems in evaluating the success of intervention strategies and in assessing the effectiveness of behavioral changes in the human population [80]. In contrast, assumptions made to reduce complexity bring major drawbacks also introduce uncertainty, with respect to overall predictive power. Infection with *V. cholerae* O1, the primary pandemic serogroup, results in protective immunity (i.e., vibriocidal antibodies) that decrease the risk of future infection [88]. Modeling cholera vaccines usually require an assumption that vaccinated individuals share the same protective rate as those naturally infected, and therefore the vaccinated susceptible individuals are treated as ‘resistant’ [89]. Age has been shown to be important, as children under the age of 5 and the elderly have the highest disease burden of cholera [90,91], but models typically assume that the population age is constant. Attempts to integrate human behavior into the SEIR model typically include the assumption that humans will behave rationally in response to the disease [83]. In practice, this assumption requires the susceptible population to be adequately informed regarding the severity of the cholera outbreak, take necessary measures to reduce contact with contaminated water/food, such as boiling or filtering water, cleaning food preparation areas, and cooking food (especially seafood) properly, and exercise appropriate sanitation practices, including the appropriate disposal of excreta. The assumption of rationality is associated with SEIR models, but it is challenging to quantify the inherent characteristics of human behavior to achieve a realistic representation of the human component in the model. While this assumption eases the model sophistication, it fails to capture the heterogeneities of cholera transmission, increasing the difference between reality and model prediction [80]. Moreover, elucidation of the human factor in cholera modeling is aggravated by the assumption that the mixing of susceptible and infectious individuals will be homogeneous. However, in reality, it has been observed that individuals move within a strong influence of socio-economic factors [92,93,94]. Hence, various methods have been used to avoid the problematic assumption of homogenous mixing, which includes dividing the susceptible population into low- and high-risk groups or into various categories, based on age, neighborhood, and behavioral risk [86,95]. In addition to these factors, education is considered to be the most cost-effective intervention strategy to prevent the transmission of cholera within at-risk populations [96].

**Table 1:** Cholera prediction using variants of susceptible-infectious-recovered models.

Author(s)	Study Descriptions/Methodology	Important Findings and Outcomes
Codeço 2001 [18]	Proposed mathematical model to explain the dynamics of epidemic and endemic cholera. This study is one of the first applications of the SIR model for cholera transmission.	Cholera epidemiology depends on social and environmental factors. Complex interaction between host and pathogen is difficult to model.
Wang et al., 2015 [83]	Separated ordinary differential equation (ODE) and reaction-convection-diffusion partial differential equation (PDE) models to examine the homogeneous and heterogeneous environments associated with cholera transmission.	Basic reproduction number (R_0_) remains a sharp threshold for disease dynamics even when human behavior is considered. Proposed mathematical justification of several consequences associated with human behavior.
Meszaros et al., 2020 [84]	Proposed a mathematical model for cholera incorporating transmission within and between households.	Vaccine interventions appeared more effective than water treatment or antibiotic administration to control household cholera.
Abrams et al., 2013 [85]	Developed three cholera surveillance models to forecast the expected number of cases in Haiti during the 2010–2011 cholera epidemic.	Models increased in complexity as more information became available: first projection estimated 105,000 cholera cases the first year; subsequent projections using different methods estimated up to 652,000 cases. Timely and realistic projections are crucial in areas with limited resources: real-time projections allowed public health officials to plan and implement response measures better.
Torres et al., 2018 [82]	Proposed and analyzed a SITRV (susceptible-infectious-treated-recovered-vaccinated) type model for cholera.	The SITRV type model fits well for the cholera outbreak in Yemen April 2017–2018. The model provides important conclusions concerning vaccination campaigns during a cholera outbreak.
Che et al., 2020 [86]	Used a “fitted” demographic equation (i.e., disease-free equation) to capture total population and a fitted low-high risk structured cholera differential equation model to study reported cholera cases in Cameroon 1987–2004.	Dual strategies of either vaccination and treatment or vaccination and improved sanitation or combined strategy of vaccination, treatment, and improved sanitation reduce the basic reproductive number of cholera cases. Rates of scaled contact and the vaccination of susceptible populations are important parameters for cholera prediction.
Dangbé et al., 2018 [87]	Proposed a model considering climatic factors and human behavior on the spread of cholera	The transmission and spread of cholera can be affected by climatic factors, the proportion of malnourished individuals, and the number of individuals practicing proper hygiene. Disease-free equilibrium stability depends on the basic reproduction number (R_0_).
Baracchini et al., 2017 [56]	Proposed a stochastic, rainfall–temperature driven model to examine the seasonality of cholera in Bangladesh.	Rainfall buffers disease transmission in wet regions while enhancing cholera resurgence in dry regions. Local variation of temperature and rainfall can be used to explain seasonal patterns.
Koepke et al., 2016 [97]	Proposed a predictive ‘susceptible-infected-recovered-susceptible’ (SIRS) type model in the form of continuous-time hidden Markov states to estimate the contribution of water depth and water temperature on the spread of cholera.	Hidden states can be used to predict an increase in infected individuals weeks before the observed number of cholera cases increases, thereby providing early notification of the epidemic. Added support to the hypothesis that environmental forces influence the trigger of a cholera outbreak.
Perez-Saez et al., 2017 [58]	Proposed a probabilistic spatial model to investigate the role human mobility plays in cholera transmission.	With respect to cholera risk, highly populated urban centers are more sensitive to El Niño/Southern Oscillation than rural periphery. Cholera risk is largely transmitted from a climate-sensitive core to the periphery. Included human mobility as a model parameter to improve outbreak prediction performance.

While these methods help to improve the prediction of cholera transmission within well-mixed populations, they fail to capture cholera modes of infection via indirect routes, such as pathogen movement via the environment, or heterogeneities relevant to disease transmission. Interaction between environment and humans is of paramount importance for predictive modeling of cholera [87]. Traditional SEIR models are less successful in dealing with indirect modes of cholera transmission, most likely explaining why they are successful in predicting highly infectious human pathogen spread via direct human-to-human contact (e.g., for viruses causing influenza and coronavirus disease 2019, COVID-19) compared to cholera, where indirect transmission plays a more important role [80]. The importance of indirect transmission routes has encouraged the incorporation of water quality models, seasonality, and climate-driven concepts into SEIR models [58,97,98,99,100]. Seasonality is more often analyzed in regions prone to flooding after heavy precipitation, such as Bangladesh [57] and Yemen [101], where monsoons promote a bimodal peak of reported cholera cases [13,17]. The integration of environmental variables into SEIR models will therefore be expected to yield better performance. Given the complex nature of *V. cholerae* in the aquatic environment, an individual environmental variable is insufficient to capture the indirect mode of cholera transmission. That is, to understand the complete dynamics of a cholera outbreak, predictive models need to capture both the direct, namely pathogen movement via humans, and indirect transmission routes. In contrast to SEIR models, models relying solely on the variability of environmental factors have shown remarkable success in real-time cholera prediction [102], suggesting that indirect transmission routes also must be monitored. Furthermore, sensitivity analysis has shown parameter uncertainty in SEIR models, implying small uncertainties in the model parameters (e.g., infection rate) may result in large variations in overall performance [80,103]. Due to parameter uncertainties with respect to cholera and an inability to incorporate indirect transmission routes, SEIR models have been less successful in modeling cholera than other infectious diseases.

## 4. Discussion

Climate variability has had a dramatic impact on marine animal and plant communities, as well as marine prokaryotes, all of which play fundamental roles in maintaining life on Earth. Over the past half-century, changes in precipitation and temperature [104] have promoted the emergence and re-emergence of infectious diseases globally [105]. The Fourth Assessment Report of the Intergovernmental Panel on Climate Change (IPCC) suggested that the world will experience enhanced climate variability, including long-term increases in precipitation, temperature, and the number of extreme events, including droughts, floods, hurricanes, and tornadoes [106]. The complex interactions between and among various environmental conditions influence the ecological niche of disease agents. For example, a number of studies have documented a pattern of poleward spreading of *V. cholerae,* demonstrating geographic expansion [21,107,108,109]. Historically, only *V. cholerae* serogroup O1 was associated with pandemic cholera. However, non-O1 *V. cholerae* are causative agents of sporadic, yet significant, infections ranging in severity from mild to life-threatening. It has recently been reported that *V. cholerae* non-O1 infections are on the rise and represent one of the most striking examples of emerging human diseases linked to climate change [109].

On both local and global scales, climate variability has the potential to significantly affect the emergence, distribution, and prevalence of infectious disease agents and thereby impose a significant burden on public health [110]. One such observation was the massive cholera outbreak in Haiti during the months following Hurricane Matthew [9]. More recently, a cholera outbreak occurred in Yemen following civil unrest in 2016. Cholera appears to be transitioning towards endemicity in that country. In both Haiti and Yemen, cholera occurred during anomalously high temperatures and precipitation, lending support to the trigger hypothesis. Despite the advances made to date, an effective cholera predictive modeling system capable of effectively capturing the transmission of disease through compartmental models has yet to be developed. The transition of cholera from epidemic to endemic in Haiti and Yemen underscores the urgent need for environmental quantitative risk models. On a global scale, it will be necessary for future models to incorporate comparative data baselines with real-time data to improve model output and prediction.

Since it is now well established that *V. cholerae* is ubiquitous in the aquatic environment and plays a critical role in nutrient cycling and in environmental homeostasis, cholera cannot be eradicated, but it can be successfully controlled. Predictive models for cholera risk assessment will be critical in the future to safeguard public health. There is much greater interest in predictive modeling, and the transmission components of such models are receiving greater attention. However, emphasis on trigger components is needed to improve our understanding of the dynamics and progression of cholera. Trigger components have been described that improve quantitative risk modeling as well as disease intervention measures. Yet, most studies of cholera transmission are mechanistic and employ compartmental models, namely the susceptible-exposed-infectious-recovered model. The limitation of mechanistically driven compartmental models is their inability to quantify the uncertainty associated with the spread of cholera. The evolution of the quantitative risk modeling associated with the trigger module is highly promising, but greater success is expected with improved transmission modeling for quantitative risk prediction. With new information from satellite remote sensing, a comprehensive transmission component for the reliable and timely prediction of cholera will surely be available in the near future.
